# From Recognition to Response: Resistance–Effector Gene Interactions in the *Brassica napus* and *Leptosphaeria maculans* Patho-System

**DOI:** 10.3390/plants14030390

**Published:** 2025-01-27

**Authors:** Zuhra Qayyum, William J. W. Thomas, Junrey C. Amas, Maria Pazos-Navarro, Jacqueline Batley

**Affiliations:** School of Biological Sciences, University of Western Australia, Perth, WA 6009, Australia; zuhra.qayyum@research.uwa.edu.au (Z.Q.); william.thomas@uwa.edu.au (W.J.W.T.); junrey.amas@uwa.edu.au (J.C.A.); maria.pazosnavarro@uwa.edu.au (M.P.-N.)

**Keywords:** plant–microbe interaction, disease, blackleg, resistance genes, *Brassica napus* L.

## Abstract

Blackleg disease, caused by the hemibiotrophic fungal pathogen *Leptosphaeria maculans*, poses a serious threat to *Brassica* crops and requires a broad understanding of the plant defence mechanisms. The *Brassica. napus*-*L. maculans* pathosystem provides a useful model to understand plant resistance response to hemibiotrophs. This review aims to explain the mechanisms underlying *R-Avr* interaction, signalling cascades, and the hypersensitive response (HR) produced by *B. napus* towards *L. maculans*, causing local cell death that restricts the pathogen to the site of infection. The role of transcription factors is pivotal to the process of HR, coordinating the regulation of genes involved in pathogen recognition and the activation of SA responsive genes and production of secondary metabolites. The *R-Avr* interaction signalling cascade involves production of reactive oxygen species (ROS), calcium ion influx, Salicylic acid (SA) hormonal signalling and mitogen activated protein kinases (MAPKs), which are critical in the HR in *B. napus*. The in-depth understanding of molecular signalling pathway of the *R-Avr* interaction between *B. napus*-*L. maculans* pathosystem provides valuable information for future research endeavours regarding enhancing disease resistance in *Brassica* crops.

## 1. Introduction

Canola (*Brassica napus* L.) is a vital oilseed crop of both economic and nutritional significance worldwide, with Canada, Europe, China, India, and Australia as its leading producers [1]. The amphidiploid genome (AACC) of canola emerged from a natural hybridization event between *Brassica rapa* (AA genome, 2n = 20) and *Brassica oleracea* (CC genome, 2n = 18). Its widespread utilisation in food, feed, and biofuel has led to heightened demands on cultivation [2]. However, sustainable canola production faces significant challenges due to the prevalence of severe fungal diseases such as blackleg, clubroot, and sclerotinia stem rot, posing threats to canola cultivation globally [1]. In *B. napus*, *Leptosphaeria maculans* incites blackleg, also referred to as phoma stem canker, which stands among the most detrimental diseases affecting this crop across Australia and Europe. The disease can cause annual loss of 10–15%, while in some cases the yield loss reaches 90% [1]. *L. maculans*, a hemibiotrophic apoplastic fungal pathogen classified within the Dothideomycetes, predominantly targets *Brassica* crops [1]. The infection process begins with the germination of ascospores that enter the leaves through the stomata and wounds, then colonising the apoplastic space of the foliar mesophyll cells and causing necrotic lesions. Within the leaves, the fungus produces asexual pycnidiospores that further increase the severity of the infection. The disease symptoms appear on the cotyledons and leaf in the form of chlorotic lesions that surrounds the infection site, while the pycnidia appear black on the surface [3]. In the mature plant, the fungus causes stem blackening, eventually killing the plant by potentially blocking the nutrient flow [4].

The impact of blackleg disease is reduced through a combination of cultural, chemical, and genetic control methods [5]. Cultural practises include rotation with other crops, stubble removal and tillage to avoid growth of *L. maculans* spores. Chemical control includes the application of fungicides. Fungicides are applied either as fertiliser-amended seed dressing or through spraying [6]. Genetic control involves growing the canola varieties with resistance to the *L. maculans* isolate [7]. Genetic resistance has been observed to be more effective in fighting blackleg disease in canola. The two types of genetic resistance in *B. napus* include qualitative (major gene) and quantitative (minor gene, polygenic, adult plant) resistance [8]. Comparatively speaking, qualitative resistance is better understood and used more frequently than quantitative resistance in *B. napus* against *L. maculans* [9]. Qualitative resistance occurs in a gene-for-gene fashion, whereby a single resistance (*R*) gene in the host interacts with a corresponding effector gene (*Avr*) in the pathogen [10]. Although there are different definitions of *R* genes, the term *R* gene is conventionally described as a single/major locus that provides highly specific resistance to the plant [11].

The plant defence responses are commonly divided into two immune pathways, pattern-triggered immunity (PTI) and effector-triggered immunity (ETI) [12]. During PTI, the pathogen is recognised by pattern recognition receptors (PRRs) that recognise the conserved pathogen associated molecular patterns (PAMPs) or microbe associated molecular patterns (MAMPs). MAMPs mostly include the microbial nucleic acids, lipoproteins, and carbohydrates, which are only produced by the microbes and not part of the host plant [13]. The PRRs are extracellular membrane receptors such as receptor-like proteins (RLPs) and receptor-like kinases (RLKs) [14]. The recognition of foreign pathogen molecules by the PRRs activates a defence response that alerts the host of an infection [15]. To cope with PTI and suppress host resistance, pathogens produce effector proteins to invade the plant causing disease, leading to effector-triggered susceptibility [16]. Previously, any carbohydrate, glycoprotein, protein, or products of the secondary pathways from the microbes were considered to be effectors [17]. However, current studies suggest that effectors are proteins that have no evolutionary conservation and control the host cellular machinery [18]. The main function of an effector protein is to help the pathogen obtain nutrients from the host [19]. The effectors are translocated into plant cells where they regulate plant immunity and spread the pathogen infection [20]. Plants have evolved specialised resistance (*R*) genes to respond to these effector molecules and provide resistance through effector triggered immunity (ETI) [16].

The pathogen recognition receptors in ETI are mostly nucleotide-binding leucine rich repeats (NLRs), and none of the cloned blackleg *R* genes are NLRs. Two of the cloned *R* genes *LepR3* and *Rlm2* are receptor like proteins (RLPs) [21] while the other three *Rlm4*, *7*, *9* are wall-associated kinase like (WAKL) resistance genes [22]. There is evidence that the PRR perception of PAMPs overlaps with the RLP detection of apoplastic fungal effectors. This is shown by the interaction of Cf4 with *BAK1* upon induction by *Avr4* in tomato during *Cladosporium fulvum* infection [23]. Another transmembrane receptor, *SOBIR1* (*SUPPRESSOROFBIR1-1*), is required for ETI initiated by RLPs [24]. It has been shown that *LepR3* and *Rlm2* require *SOBIR1* for initiating the plant defence response in *B. napus* [25]. This kind of effector triggered defence response generated by RLPs is also called an RLP-triggered incompatible (resistant) interaction or effector triggered defence (ETD) [26]. During an incompatible interaction, the host *R* gene matches with the pathogen *Avr* gene and initiates a hypersensitive response (HR), killing the cells surrounding the infection site and preventing the spread of infection [27]. On the other hand, if the pathogen successfully infects the plant in the absence of the corresponding *R* gene, a compatible (susceptible) interaction takes place with the development of disease in the plant [28].

ETD is the defence response of plants against apoplastic fungal pathogens, and it does not eliminate the fungus completely. It slows the sexual reproduction cycle of the fungal pathogen and therefore the term defence is more appropriate than immunity [29]. The effectors of apoplastic pathogens are recognised at the cell surface and the receptors that initiate ETI or ETD differ in their protein domain, as well as in their interaction with other signalling partner proteins [30]. The receptors in ETD are RLPs that span the plant membrane and contain an extracellular leucine rich repeat domain and a short cytoplasmic tail without a signalling motif [31,32]. Further research is required to study the hypersensitive response produced by *B. napus* against *L. maculans* to be considered ETD. The *R* (*LepR3* and *Rlm2*) genes encoding RLPs that have been cloned in *B. napus* against the apoplastic pathogen *L. maculans* could be useful in understanding the ETD in *B. napus* [33]. The signalling pattern observed during defence response activated by these cloned genes could be used to understand whether the immunity in *B. napus* is ETI or ETD.

Pathogen recognition is followed by the activation of mitogen-activated protein kinases (MAPK), closure of stomata, regulation of defence-responsive genes, pathogenesis-related genes, callose deposition, and production of reactive oxygen species [34]. Subsequently, the large-scale transcriptional reprogramming initiates phytohormonal signalling during pathogen infiltration; specifically the activation of ethylene, jasmonic acid (JA), and salicylic acid (SA) during incompatible interaction of *L. maculans* in *B. napus* [35]. SA, JA, and ethylene are the major signalling phytohormones in plants, and they are activated in PTI, ETI, and ETD [36,37]. An understanding of the plant pathogen interactions leading to resistance is a prerequisite for the functional characterisation of genes involved in the interaction. Moreover, it also helps to develop long-term strategies to control disease progression in plants [38]. This review aims to elucidate the intricacies of the *R-Avr* interaction during *B. napus-L. maculans* interactions, offering foundational insights essential for advancing our comprehension of plant immunity.

## 2. Dynamics of Fungal Genes During Host Infection

The regulation of genes plays a crucial role in controlling the hemibiotrophic nature of a fungus by orchestrating its transition between the biotrophic and necrotrophic phases during infection. RNA sequencing (RNA-seq) allows the entire transcriptome of an organism or tissue to be profiled, providing insights into which genes are actively transcribed under specific conditions. Differential gene expression analysis between different conditions (e.g., normal vs. disease state, different developmental stages) can reveal key regulatory pathways and transcription factors involved in disease progression [39]. Gene expression studies of the interaction between *L. maculans* and *B. napus* can help us better understand the processes underlying the *R-Avr* interaction [40]. The compatible and incompatible interaction studies between *Rlm1-AvRlm1* [41] and *Rlm2-AvRlm2* [42] provides insight into how the fungus regulates its genes to initiate the infection process. Disease progression in plants occurs through the enzymatic degradation of proteins and carbohydrates from the plant cell wall through a specific class of enzymes, CAZys (Carbohydrate-degrading enzymes), secreted by the fungus [42]. These enzymes have well-characterised domains, and apart from providing nutrition, the carbohydrate-degrading activity releases products that act as damage-associated molecular patterns (DAMPs) which activate host immunity [43]. The most frequently expressed CAZys during the *Rlm1-AvRlm1* incompatible interaction were *CBM* (carbohydrate-binding module) and pectate lyases. CAZys are highly expressed during the initial days of infection in both compatible and incompatible *B. napus*–*L. maculans* interaction, and decrease afterwards during the incompatible interaction, indicating the suppression of the fungus by the plant [42]. CAZy genes facilitate the fungal progression through the plant’s intercellular spaces during the biotrophic stage of *L. maculans* infection [44]. This indicates that *L. maculans* rapidly adjusts its CAZyme expression upon encountering different hosts, suggesting an adaptive response to host-specific defences. Targeted gene silencing of CAZys in the pathogen can help to determine the effectiveness of these enzymes in disease onset.

Fungal pathogen effectors are also known as avirulence (*Avr*) proteins which help in disease onset by interfering with host immunity and/or modifying host cellular activities [45]. Twelve *Avr* genes from *L. maculans* have been cloned and characterised. This has established the *L. maculans*–*Brassica* system as an exemplary model for comprehending host–pathogen interactions [46]. *L. maculans*, being a hemibiotroph, will initially rely on effectors to suppress plant defence, and then will subsequently use effectors to kill plant cells. In *L. maculans*, most putative or candidate effectors are localised in transposon-rich repetitive DNA and are affected by repeat-induced point mutations [3]. The host–pathogen interaction in *L. maculans* often leads to genetic variation in *Avr* genes that helps to overcome disease resistance in host plants, resulting in the rapid breakdown of resistance [47]. The effector genes are located in the transposable rich element region and are prone to mutation on a large scale. The loss of function of *AvrLm3* to overcome *Rlm3* resistance [47] shows that any environmental stress threatening the pathogen fitness might trigger the mutations in the *Avr* gene [48]. The pathogen evolution is one of the main challenging factors in providing a long-term solution for disease resistance.

The protein obtained from plant cell walls is a potential source of nitrogen for the fungus. To release protein from the plant cell wall, a trypsin-like peptidase was highly expressed during the *Rlm1-AvRlm1* and *Rlm2-AvRlm2* interactions in *B. napus* [44]. The increased expression of protein and carbohydrate degrading enzymes during incompatible interaction supports their role in fungal progression in *B. napus*. Moreover, the genes involved in necrosis and ethylene induction also showed higher expression during the pathogenic phase, causing necrotic symptoms in the plant [42]. Polyketide synthase (PKS) was activated in the fungal genome, facilitating its growth [41]. GT21, which encodes biosynthetic enzymes involved in glycosylation of lipids, and CE12 encoding pectin acetyl esterase, were also highly upregulated in *L. maculans*. Moreover, the expression of genes encoding chitinases also increased 2 days post inoculation (dpi), as they help the fungus in growth and development [49]. The expression of genes controlling sirodesmin production was lower during the initial stages of infection, and then slightly increased towards 11 dpi. Sirodesmin PL is a non-host-selective phytotoxin produced by *L. maculans*, [50]. Host-induced gene silencing and RNA silencing is an effective approach to understand the elaborate role of these genes in fungal disease progression [51]. It will also help to prevent the fungal progression in the host plant. Pectate lyase *PL3*, cytochrome oxidase P450, *Lm5LysM* are some other genes expressed during early stages of infection involved in protein degradation and fungal colonisation [44]. By analysing the dynamics of gene expression over time, researchers gain insights into the molecular mechanisms underlying host susceptibility or resistance, which can help develop strategies for enhancing crop resistance against pathogens. This could be performed through the transcriptome profiling, transcriptional regulation studies, and system biology approaches.

## 3. Pathogen Recognition-Mediated Host Resistance

**Figure 1:**
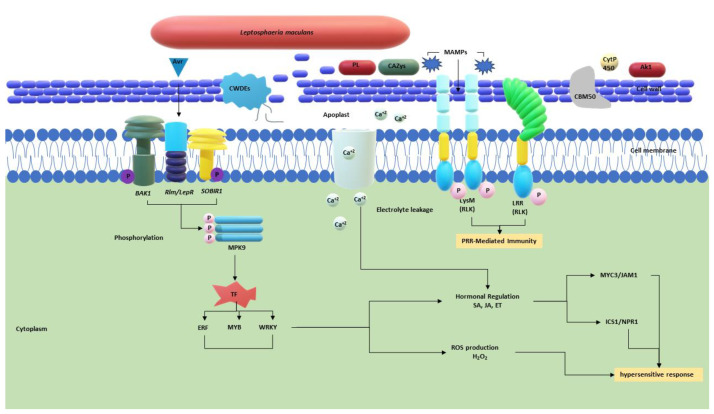
Schematic diagram of the potential signalling and regulatory gene network during the *R-Avr* interaction in *Brassica napus*–*Leptosphaeria. maculans* pathosystem producing a hypersensitive response. The arrows indicate the pathways and the regulatory factors activated during the immune response. Abbreviations: CAZys, Carbohydrate active enzymes; CWDEs, Cell wall-degrading enzymes; PL, Pectin lyases; SA, Salicylic acid; JA, Jasmonic acid; ET, Ethylene; ICS1, Isochorismate synthase 1.

So far, 21 blackleg *R* genes have been genetically mapped in *B. napus*, *B. rapa*, and *B. juncea* (Table 1). However, only five of these *R* genes, *LepR3*, *Rlm2*, *Rlm4*, *Rlm7*, and *Rlm9*, have been cloned in *B. napus* [21,22,32,52]. Among the five cloned genes, *LepR3* and *Rlm2* are allelic variants of the *LepR3* LRR-RLP locus, recognising *AvRlm1-AvrLep3* and *AvRlm2*, respectively [21]. While the other three cloned genes *Rlm4*, *Rlm7* [22], and *Rlm9* [52] are WAKL proteins. Differential gene expression studies of the interaction between *LepR3-AvRlm1* and *Rlm2-AvRlm2* showed that several receptor kinase genes were upregulated. The differentially upregulated receptor kinase genes included RLPs, cysteine-rich RLKs (cysRLKs), leucine-rich receptor like kinase (LRR-RLKs), WAKLs, and RLKs [53]. Similar results were observed during the transcriptome profiling of *LepR1* resistant [54] and *Rlm1* resistant *B. napus* [41]. *LepR3* encodes an RLP that interacts with *AvRlm1* to initiate an HR, providing complete resistance [32]. RLPs have a signal peptide that helps them facilitate the movement towards the cell membrane, a transmembrane domain, a short extracellular domain that has multiple leucine rich repeat (LRR) motifs involved in protein–protein interaction and a short intracellular fragment [55] (Figure 1). The expression of RLP supports the ETD response in *B. napus* as proposed by Stotz [26].

In the blackleg pathosystem, most of the *R* genes interact with a single *Avr* gene following a gene-for-gene interaction, whereby each *R* gene recognises a corresponding *Avr* gene in the pathogen. For example, *Rlm2* interacts with *Avrlm2*, *LepR1* with *AvrLep1*, and *Rlm6* with *Avrlm6* [31,56,60]. However, recent studies have shown that some *R-Avr* gene interactions are more complicated and involve multiple genes. One such example is the recognition of *Avr10A* and *Avr10B* by *Rlm1*0. Complementation and gene silencing assays showed that both *Avr* genes are required to trigger the necessary *Rlm1*0 resistance. In vitro and in planta studies confirmed that both *Avrlm1*0 genes interact physically with *Rlm1*0 [62]. The avirulence gene *AvRlm1* is recognised by both *LepR3* and *Rlm1* [32,68]. Apart from these *R* genes, some other components of the PAMP/effector receptor complexes have been found to be expressed during early stages of *L. maculans* infection [40]. Overall, the activation of several receptorssuch as RLPs, cysRLKs, WAKLs, and LRR-RLKs during incompatible interaction provide the basis for further studies to confirm the nature of plant immunity in *B. napus*.

Interactome analysis of *LepR3* with *Avrlm1* showed that the effector molecule was limited to the extracellular space. The interaction took place extracellularly, providing evidence that *LepR3* is a cell surface receptor and that the C-terminal region of *Avrlm1* is required for the interaction with *LepR3* [25]. Transcriptome profiling of the resistant *B. napus* identified another LRR-RLK, *SUPPRESSOR OF BIR1-1 (SOBIR1)* [54] that interacts with RLPs to initiate ETI [24]. *SOBIR1* was initially identified as a suppressor of *BIR1* (*BAK1*-interacting receptor-like kinase 1) and is conserved across the plant kingdom. It is required for *Cf2*-, *Cf4*-, and *Ve*1-mediated HR in tobacco and resistance of tomato against the fungal pathogens *C. fulvum* and *Verticillium dahliae*, respectively [24,69]. The kinase domain of *SOBIR1* is suggested to be involved in downstream signalling when interacting with *Cf4/Avr4* complex, however, it does not only respond for interaction with *Cf4* [70]. Two *B. napus* orthologs of *SOBIR1*, *BnSOBIR1-A3* and *BnSOBIR1-C3*, have been successfully cloned and utilised for their interaction analysis between *LepR3* and *Avrlm1*. The study showed that *LepR3* interacts with *BnSOBIR1* to initiate HR [25]. This is further strengthened by the fact that when *NbSOBIR1*, which shared sequence homology with *BnSOBIR1-A3*, was silenced in *Nicotiana benthamiana* plants, the HR was compromised [24]. Recent studies have also reported the role of *BRI1*-Associated Kinase 1 *(BAK1)/SOMATIC EMBRYOGENESIS RECEPTOR KINASE (SERK)3* in *R-*gene mediated resistance [71]. To confirm the role of *BAK1* in programmed cell death during the *LepR3/AvRlm1* interaction, a mutant analysis was performed by transient gene expression in *N. benthamiana* plants. The co-expression analysis of *NbSER3a/b* knockout constructs, along with the *LepR3/Avrlm1* interaction, showed that the plant was not able to produce a HR [25]. This indicates that *LepR3* forms a complex with *SOBIR1* and *BAK1* to initiate HR, causing programmed cell death. However, the exact mechanism of interaction needs further study to determine whether the proteins undergo phosphorylation or ubiquitination to form the receptor complex. Moreover, whether all the *R* genes form a receptor complex with other RLKs, or if some interact independently to initiate immunity, remains to be determined. This could be performed with the identification and cloning of more *R* genes that would help an understanding of the *B. napus* response to *L. maculans*.

## 4. Genetics of Transcriptional Reprogramming

Transcription factors (TF) have emerged as pivotal regulators of stress-responsive genes, positioned as promising candidates for enhancing crop resilience [72]. Transcriptome profiling of *B. napus* infected with *L. maculans* showed that the transcription factors mostly involved in regulating the expression of defence-related genes were upregulated. This includes ethylene responsive factors (ERFs)/Apetala2 (AP2)-domain, basic leucine zipper (bZIP), MYB, WRKY, and transcription factors with a zing-finger binding domain (ZF-BD). WRKY transcription factors play an important role in plant defence responses and physiological processes and are plant specific. A total of 46 WRKY TFs have been identified with 38 cloned in *B. napus*, conferring resistance against *L. maculans* infection [73]. The expression of WRKY 28 in *B. napus* indicates that the plant also responds to the pathogen infection by regulating SA biosynthesis. WRKY 28 is involved in the transcriptional activation of the SA biosynthetic enzyme iso-chorismate synthase 1 (*ICS1*) [74]. Plant-specific WRKY TFs are a class of DNA binding proteins and are actively induced when the plant is exposed to biotic and abiotic stress, including SA and other molecules. Moreover, the expression of WRKY TF is rapid, transient, and tissue-specific [75]. WRKY TFs have a DNA binding domain characterised by a highly conserved WRKYGQK motif [76] and a Zn-chelating domain containing Zn finger motif [77]. They inhibit or activate the transcription of the genes involved in physiological responses [78].

The WRKY TF plays a positive and negative regulatory role in the biotic defensive response, organising a complex network of defence mechanisms against pathogen invasion. Phosphorylation of *AtWRKY33* by *MAPK3/6* increases disease resistance through phytoalexin biosynthesis [79]. It is suggested that the WRKY TFs positively regulating the disease resistance may directly activate the transcription of the resistance gene. One such example is the binding of the WRKY DNA binding domain to the W box of the promoter region of *Arabidopsis Natriuretic peptide receptor 1 (NPR1)*. On the other hand, *WRKY25* negatively regulates the expression of SA-mediated defence response, while positively regulating ET biosynthesis in resistant *B. napus* [80]. Several WRKY TFs are reported to be involved in the MAPK signalling pathway for the regulation of plant resistance response towards pathogens as substrates of MPK [81]. *AtWRKY46* is identified as a substrate of *AtMPK3* through in vitro phosphorylation [82]. The negative regulation of the defence response by WRKY needs further study to determine their role in *B. napus* ETI as several WRKY TFs are activated in the incompatible interaction.

Similarly, ERFs, MYB, WRKY, and members of the NAC transcription factor family were upregulated during the interaction between *LepR3*-*Avrlm1*, *Rlm2*-*Avrlm2*, *LepR1*-*AvrLep1*, and *Rlm1*-*Avrlm1*. NAC and MYB TFs families are involved in lignin biosynthesis [83,84]. Gene enrichment analysis also identified several factors involved in protein ubiquitination, protein post-translational modification and the deployment of pathogenesis-related protein kinases [53]. The NAC and WRKY transcription family were actively upregulated during *LepR1*-*AvrLep1* incompatible studies, as compared to compatible *B. napus*–*L. maculans* studies [54]. The overexpression of *BnNAC19* in B. napus provides enhanced resistance towards the *L. maculans* [85]. The APETALA2/ERF transcription family plays an important part in biotic stress, wounding, cold, drought, heat, and salinity stress [86]. Ethylene-responsive factors *ERF054* and *ERF053* are upregulated during the *L. maculans* infection in *B. napus*, indicating their role in biotic stress response [53]. Members of the MYB transcription family have been studied in Arabidopsis for functional characterisation, which shows that they are involved in regulatory networks controlling development, metabolism, and responses to biotic and abiotic stresses [87]. Several MYB transcription factors were upregulated during the *L. maculans* infection on *B. napus* [53].

The upregulation and downregulation of MYB transcription factors are quite critical to the defence signalling during the *L. maculans* attack. However, the detailed mechanism of action is not explained very well and needs further study in *B. napus*. The study of *AtMYB30* induced HR in *A. thaliana* when infected with the biotrophic fungus *Cercospora nicotianae.* The overexpression and mutation of *AtMYB30* in *A. thaliana* and *N. benthamiana* resulted in typical HR and increased diseases symptoms, respectively, following fungal infection [88]. Several MYB TFs are directly involved in defence response to biotic stress by regulating the JA signalling pathways. For example, *AtMYB44* overexpression inhibits the JA signalling and impairs defence response to *E. carotovora* [89]. *CsMYB96*, a homologue of *AtMYB96*, enhances the expression of the SA biosynthesis gene resulting in host defence to *Botrytis cinerea* [90]. However, some MYB TFs such as *GhMYB33* negatively regulates the plants defence response. The silencing of *GhMYB33* increases the defence response to *V. dahiliae* [89]. Despite the crucial role of MYB TFs in plant defence responses, the underlying molecular mechanisms are not completely explored. As plants initiate the activities of several MYB TFs during biotic stress, it is unclear how these TFs differentiate between the different stresses and initiate the correct response [89]. A similar study in *B. napus* with overexpressed and mutated MYB TFs could help determine their role in HR.

Some other notable transcription factors expressed during the interaction are heat-responsive B-4-like, bHLH, GATA TF 3, and JUNGBRUNNEN 1 TFs [53]. Most of the transcription factors are activated early in the infection process which suggests that the timely activation of TFs is essential for cellular reprogramming in early defence response [54]. TFs play a critical role in *B. napus* response to *L. maculans* by carefully regulating gene expression and providing disease resistance. Understanding the role of individual TFs in disease resistance not only provides insight into the mechanism of interaction but will help to improve the overall immunity in *B. napus* against *L. maculans* infection.

## 5. Activation of Genes Involved in Resistance Mechanism and Hormonal Regulation

The genes involved in cell death are specifically upregulated in the resistant *B. napus* during the initial stages of infection (Table 2). These genes include *BAX INHIBITOR 1*, *DEVELOPMENT AND CELL DEATH 1*, *NUDIX HYDROXYLASE HOMOLOG 7*, *METACASPASE 2*, *NECROTIC SPOTTED LESIONS 1*. The activation of these genes during the early stages of infection limits the spread of lesions during the biotrophic-necrotrophic transition of *L. maculans*. Sulphur and its related compounds (SDCs) glutathione, glucosinolates, and phytoalexins, are involved in disease resistance in plants [91]. SDCs are involved in perception of pathogen and signal transduction to activate defence response through phytohormonal regulation (salicylic acid, jasmonic acid, and ethylene) and production of reactive oxygen species (ROS) [92]. *GLUTATHIONE SYNTHETASE 2* analogues were differentially upregulated in resistant *B. napus* at 3 dpi. Glutathione is a key component of sulphur metabolism and helps in toxin neutralisation by the activity of glutathione-S-transferases (*GST*) [54].

Glucosinolates are sulphur rich plant secondary metabolites mainly found in Brassica species that provide immunity against biotic stresses [93]. Two major IGS biosynthetic pathway genes, *CYP79B2* and *CYP83B1*, are upregulated in resistant *B. napus*, while they were highly downregulated in susceptible *B. napus* during blackleg [54]. The accumulation of glucosinolates in *B. napus* demonstrated their role in providing immunity to the plant against fungal infection. However, the exact mechanism of action for these secondary metabolites needs to be studied further.

Hormones manifest the plant responses to both biotic and abiotic stresses by directing several complicated signalling pathways. The primary hormones involved in regulating these responses are SA, JA, ethylene (Et), and abscisic acid [94]. During the incompatible interaction of *LepR3*-*AvRlm1* and *Rlm2*-*AvRlm2*, the expression of SA biosynthesis genes increased fourfold in resistant as compared to susceptible *B. napus* [53]. RNA sequencing and gene ontology enrichment analysis also showed that the genes involved in SA, JA, and Et-mediated signalling networks were upregulated in *B. napus* during the *LepR1*-*AvrLep1* interaction [54]. Similarly, the expression of SA pathway marker genes, ICS1 and WRKY70, were upregulated during the *Rlm1*-*Avrlm1* interaction in incompatible interaction [35]. The early activation of SA responsive genes shows that the resistant *B. napus* effectively slows down the pathogen life cycle. The SA and conjugated SA glucoside causes the slow release of Calcium ions and oxidative burst [95]. Calcium ion (Ca^+2^) signalling also plays an important role in *B. napus* resistance to *L. maculans*, as shown by the increased expression of calcium transporters (Cyclic nucleotide-gated calcium channel 3, 12, and 19), as well as calcium dependent signal transducers in resistant *B. napus* [28]. Therefore, the immune response is likely to be directed and amplified by calcium signalling working in combination with *Rlm2*/SOBIR1-mediated signalling.

**Figure 2:**
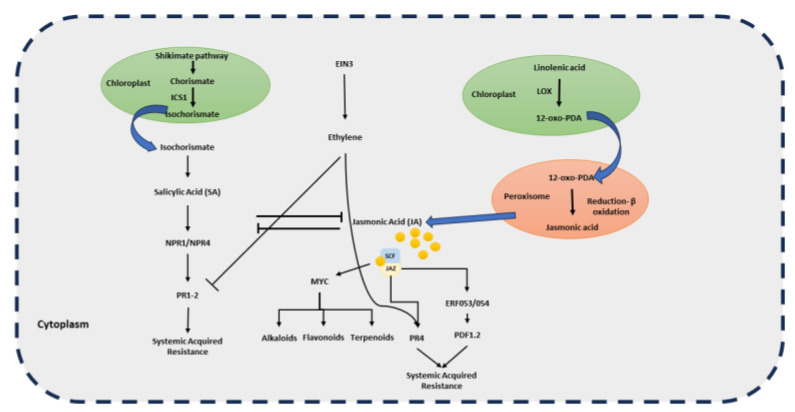
The SA and Et/JA signalling in *Brassica. napus to Leptosphaeria. maculans* infection during *R-Avr* interaction. The SA biosynthesis results in the production of the PR1 gene that produces systemic acquired resistance. However, the SA biosynthesis is repressed by the Et/JA signalling to localise the cell death at the site of infection.

The SA biosynthetic gene *ISOCHORISMATE SYNTHASE 1* and SA marker gene *PATHOGENESIS-RELATED GENE1* (*PR1*) (Figure 2) were upregulated in cells close to the inoculation site during *Rlm1*-*Avrlm1* interaction. Similarly, an increase in expression of Et/JA biosynthesis including *ACC OXIDASE* and *PDF1.2* was also observed in the resistant *B. napus* during *LepR1*-*AvrLep1* incompatible interaction. On the contrary, *LIPOXEGENASE1, ALLENE OXIDE3*, and *ALLENE OXIDE CYCLASE 3* genes are downregulated in the susceptible cotyledons [54]. JA signalling is associated with resistance against herbivory and necrotrophic pathogens. However, it is also involved in suppressing the SA signalling pathway against biotrophic and hemibiotrophic pathogens. Therefore, pathogens utilise these pathways to weaken the SA-dependent responses in plants [96]. The increased expression of JA biosynthetic genes during the initial days of infection indicates that *L. maculans* tries to suppress the disease resistance response. [97]. It shows that the early recognition of the *Avr* effector by the plant *R* gene results in the activation of genes involved in hormonal signalling [98].

The expression pattern of hormonal signalling between SA and JA/Et is unusual in the incompatible interaction in the *B. napus-L. maculans*–pathosystem [98]. The conventional hormonal signalling shows that SA and JA have antagonistic relationship. However, the earlier activation of SA, JA, and Et responsive genes could be a balanced defence signalling strategy, where SA and Et promotes the production of ROS and oxidative bursts, while JA reduces the effect of ROS-induced cell senescence [99]. The expression of SA signalling genes correlates with the biotrophic nature of the pathogen during the early stages of infection and prevents the spread of pathogen. The upregulation of SA biosynthetic genes in resistant *B. napus* emphasises the role of SA in providing immunity against the *L. maculans* by restricting it to the site of infection and preventing the transition to the necrotrophic stage.

## 6. Production of Reactive Oxygen Species

The oxidative burst is considered an important phenomenon in plant defence signalling. It involves the activation of pathogenesis related (PR) gene expression, electrolyte leakage, phytoalexin production, and programmed cell death upon defence signalling. When the fungal pathogen infects the plant, it releases elicitors that trigger the production of reactive oxygen species (ROS) such as hydrogen peroxide (H_2_O_2_), superoxide (O^2−^), hydroxyl radical (·OH), and the singlet oxygen (1O_2_). These ROS are detrimental to the fungus and direct oxidative cross-linking of the cell wall, which prevents the penetration of the fungus into the plant cells [100]. The level of H_2_O_2_ increased several folds during the *LepR3-AvRlm1* incompatible interaction as well in overexpressed *LepR3 B. napus* compared to the compatible interaction indicating their role in cell death [53]. ROS have more than one role in the plant–pathogen interaction. They prevent the spread of biotrophic pathogens by stimulating cell death, but can also damage the nucleic acid, the membranes, and proteins [101]. This damage can indirectly facilitate fungal colonisation by releasing nutrients which can be utilised by the pathogen [102]. Therefore, plants have evolved an elaborate pathway involving enzymatic and non-enzymatic scavenging systems to control the level of ROS inside the cell. Some of the well-documented components of these processes are ascorbate peroxidase (APX), peroxidase (POX), superoxide dismutase (SOD), catalase (CAT), antioxidants glutathione, ascorbic acid, tocopherol, and carotenoids [103]. Another study on the *B. napus*–*L. maculans* interaction showed an increase in the level of H_2_O_2_ in the plant cells. DAB (3,3′-diaminobenzidine) staining showed that H_2_O_2_ levels increased 6 dpi with the fungus. In vitro studies showed that the fungal spores are affected by high concentrations of H_2_O_2,_ which restricts their growth. The activity of the enzymes *GPX*, *APX*, *GR*, *CAT*, and *SOD* involved in the oxidative pathway was monitored and an increased accumulation of ROS was identified [102]. The results of increased ROS accumulation were in accordance with those of other pathogens such as the interaction between the hemibiotrophic pathogen *Septoria tritici* and wheat [104]. All these events lead to cell death, restricting the pathogen to the site of infection. The controlled production of ROS will help to improve the immunity of *B. napus* against *L. maculans* without compromising the health of the plant.

## 7. Conclusions and Future Perspectives

The increasing number of studies on the incompatible interaction between *B. napus* and *L. maculans* have provided valuable insights into the mechanisms of pathogen recognition and the plant defence response. These responses are complex and comprise many regulatory factors and genes involved in plant-pathogen interactions through PTI, ETI [41], and ETD. High-throughput genome sequencing and transcriptome analysis have helped capture significant molecular pathways with the identification of DEGs involved in *B. napus* immunity to *L. maculans*. This constitutes the activation of *R* genes as well as the production of ROS, causing necrosis of cells and limiting the pathogen to the site of infection. The knowledge obtained through these studies can help regulate plant signalling mechanisms to improve *B. napus* resistance to *L. maculans*.

Techniques for examining protein–protein interaction, such as bi-immunofluorescence complementation assays (BiFC), provide an effective approach to studying the mechanism of *R-Avr* interactions in the *B. napus-L. maculans* pathosystem [25]. However, the complexity of the *B. napus*-*L. maculans* interaction, coupled with the genetic variability of both host and pathogen, presents hurdles in unravelling the full spectrum of *R-Avr* interactions. Looking ahead, the prospects of insight into *B. napus*-*L. maculans* interaction studies are promising. Advances in high-throughput sequencing technologies, and genome editing, will enable researchers to characterise more *R* genes functionally. Moreover, the functional characterisation of *R* genes will facilitate the development of resistant *B. napus* varieties without the risk of resistance breakdown, providing broad-range protection. This is particularly important regarding the ability of *L. maculans* to evolve and adapt to the *R* gene, overcoming resistance constantly. As *B. napus* resistance heavily relies on the detailed understanding of *R* genes, the cloning of *R* genes will help the development of resistant varieties, enhancing crop resilience and ensuring global food security in the face of evolving plant diseases.

## Figures and Tables

**Table 1:** *Brassica napus R* genes and their interacting *Leptosphaeria maculans Avr* genes.

*R* Gene	R Protein	*Avr* Gene	Reference
*LepR1*	-	*AvrLep1*	[9,54,56]
*LepR2*	-	*AvrLmS-Lep2* *	[40,56]
*LepR3* *	RLP	*AvrLm1* *-Lep3	[32]
*LepR4*	-	*-*	[57]
*LepR5*	-	*-*	[58]
*LepR6*	-	*-*	[58]
*Rlm1*	-	*AvrLm1* *-Lep3 *	[59]
*Rlm2* *	RLP	*AvrLm2* *	[21]
*Rlm3*	-	*AvrLm3* *	[60]
*Rlm4* *	WAKL	*AvrLm4* *-7 *	[22]
*Rlm7* *			
*Rlm6*	-	*AvrLm6* *	[61]
*Rlm5*	-	*AvrLm5* *-9 *	[38]
*Rlm9* *	WAKL		[52]
*Rlm10*	-	*AvrLm10A* *-AvrLm10B *	[62]
*-*	-	*AvrLm11* *	[63]
*Rlm12*	-	*-*	[64]
*Rlm13*	-	*-*	[65]
*Rlm14*	-	*AvrLm14* *	[66]
*RlmS*	-	*AvrLmS-Lep2* *	[67]

* Indicates the cloned *R* and *Avr* genes.

**Table 2:** List of genes upregulated specifically during the *R-Avr* interaction in resistant *Brassica napus* during infection by the *Leptosphaeria. maculans*.

Gene Name	Function	*R-Avr* Interaction	Reference
***RLP30***	Mediate innate immunity to necrotrophic pathogen in *Arabidopsis*.	*LepR1-AvrLep1*	[54]
***BAX INHIBITOR 1***	Inhibit programmed cell death allowing cell survival,	*LepR1-AvrLep1*	[54]
***SUPPRESSOR OF BIR 1***	required for receptor-like kinase and receptor-like protein function.	*LepR1-AvrLep1*	[54]
***MKK9* Homologue **	interacts with MPKs during signalling.	*LepR1-AvrLep1*	[54]
***ISOCHORISMATE SYNTHASE 1***	SA biosynthetic gene	*LepR3*-*AvRlm1* and *Rlm1-AvRlm1*	[53]
***PATHOGENESIS-RELATED GENE 1*** **(*PR1*)**	SA marker gene	*Rlm1-AvRlm1*	[41]
***ACC OXIDASE 2***	ET/JA biosynthetic genes	*LepR3-AvRlm1*	[53]
***PDF1.2***	ET-JA marker gene	*LepR1-AvrLep1*	[54]
***LIPOXEGENASE 2*** **(*LOX2*)**	JA-biosynthetic genes (plant growth and development and responses to abiotic and biotic stresses.)	*LepR1-AvrLep1*	[54]
***ALLENE OXIDE SYNTHASE*** **(*AOS*)**	JA-biosynthetic genes.	*LepR1-AvrLep1*	[54]
***ALLENE OXIDE CYCLASE 3***	JA-biosynthetic genes (involved in production oxo-phytodienoic acid (OPDA)).	*LepR1-AvrLep1*	[54]

## Data Availability

The article has no linked data.
